# Guidelines for the Fitting of Anomalous Diffusion Mean Square Displacement Graphs from Single Particle Tracking Experiments

**DOI:** 10.1371/journal.pone.0117722

**Published:** 2015-02-13

**Authors:** Eldad Kepten, Aleksander Weron, Grzegorz Sikora, Krzysztof Burnecki, Yuval Garini

**Affiliations:** 1 Physics Department & Institute of Nanotechnology, Bar Ilan University, Ramat Gan, Israel; 2 Hugo Steinhaus Center, Institute of Mathematics and Computer Science, Wroclaw University of Technology, Wroclaw, Poland; Weizmann Institute of Science, ISRAEL

## Abstract

Single particle tracking is an essential tool in the study of complex systems and biophysics and it is commonly analyzed by the time-averaged mean square displacement (MSD) of the diffusive trajectories. However, past work has shown that MSDs are susceptible to significant errors and biases, preventing the comparison and assessment of experimental studies. Here, we attempt to extract practical guidelines for the estimation of anomalous time averaged MSDs through the simulation of multiple scenarios with fractional Brownian motion as a representative of a large class of fractional ergodic processes. We extract the precision and accuracy of the fitted MSD for various anomalous exponents and measurement errors with respect to measurement length and maximum time lags. Based on the calculated precision maps, we present guidelines to improve accuracy in single particle studies. Importantly, we find that in some experimental conditions, the time averaged MSD should not be used as an estimator.

## Introduction

The analysis of single particle trajectories has become a standard procedure in the analysis of experimental and theoretical systems [1–7]. In biological systems, that are intrinsically stochastic in nature, single particles have been measured in all cellular environments and stages, both in vivo and in vitro [8–17].

Since cellular environments are complex microscopic systems with a strong thermal component [17], the motion of single particles, even if directed, incorporates a random diffusive component, which must be characterized in order to build a physical picture of the system [18–24]. A common tool by which the diffusion of a single particle is classified is the time averaged mean square displacement (TAMSD) [14–17, 25–31]:

$$\bar{\delta^{2}}\left(\tau \right)=\frac{\sum_{m=1}^{L/\delta -n}{\left(x\left(m\delta +\tau \right)-x\left(m\delta \right)\right)}^{2}}{L/\delta -n},$$ (1)

defined here for a trajectory *x*(*t*) of length *L*, taken at sampling time-intervals *δ* and the averaging window is *τ* = *nδ*. For normal diffusion (not necessarily Brownian or Gaussian [32]) the MSD is linear in time $\bar{\delta^{2}}(\tau )=D_{1}\tau$, where *D*_1_ is the generalized diffusion coefficient which includes all constant prefactors, depending on the diffusion mechanism.

The TAMSD may be of any functional form, but in many cases it is a power law function over long times, $\bar{\delta^{2}}(\tau )=D_{\alpha }\tau^{\alpha }$ [33, 34]. The anomalous exponent *α* is related to fundamental characteristics of the stochastic process, such as temporal correlations and the distribution of particle steps and it is necessary for predicting the future particle motion, first passage times and more [35].

There are various classes of anomalous diffusion and they all result from the breaking of the assumptions behind normal Brownian diffusion, see [36] for a recent review. Continuous time random walks (CTRW) which have long tailed jump distributions or waiting times between jumps exhibit weak ergodicity breaking of a normal TAMSD. Variation in the surrounding space may lead, among other models, to heterogeneous diffusion processes (HDP) and obstructed diffusion, both with unique characteristics. If the stochastic process is not Markovian and there is a temporal correlation between steps, another class of anomalous diffusion is exhibited. Fractional Brownian motion (FBM) for example has self-similar Gaussian steps with a correlation that decays as a power law. A general description of processes with temporal step correlations can be obtained through the ARFIMA framework that generalizes fractional dynamics through a discrete generating process [37].

**Fig 1:**
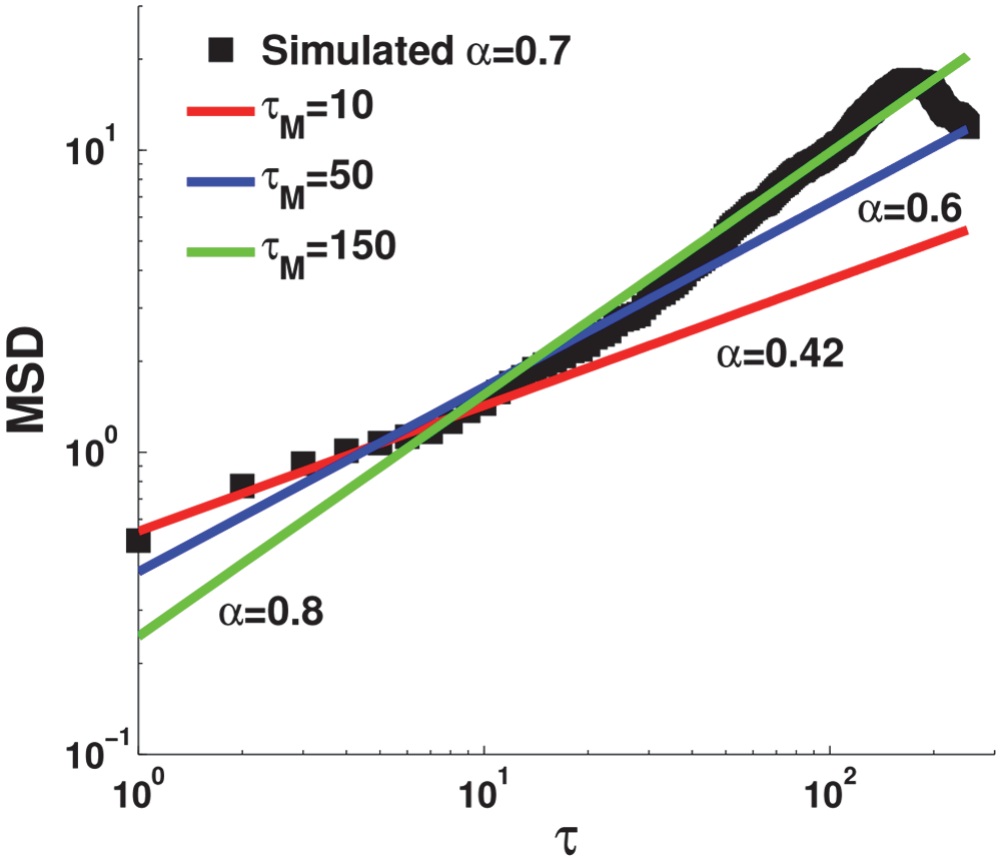
Fitting a time averaged MSD with various maximum time lags. A trajectory with *α* = 0.7, *L* = 2^9^, *σ* = 0.5 was simulated (black squares) and fitted for various *τ*_*M*_ values. While the small *τ*_*M*_ fitting (red *τ*_*M*_ = 10 and blue *τ*_*M*_ = 50) underestimated *α*, the large *τ*_*M*_ (green *τ*_*M*_ = 150) gives an overestimation. Clearly, selecting the optimal *τ*_*M*_ value is not trivial as both small and large values may lead to erroneous results. Graphically assessing the quality of the fit does not help select the best *τ*_*M*_ either.

The TAMSD is normally fitted through the logarithm of eqn. 1 as a function of *τ* up to a maximal *τ*_*M*_, Fig. 1:

$$\log\left(\bar{\delta^{2}}\left(\tau \right)\right)=\log\left(D_{\alpha }\right)+\alpha \log\left(\tau \right),\;\tau =1,\ldots ,\tau_{M}.$$ (2)

Several studies have shown that the TAMSD is a problematic estimator [25, 38, 39]. The internal correlations between the averaged quantities merit the central limit theorem inapplicable and large variations are introduced with increasing *τ*. In addition, measurement errors lead to short time artifacts in the estimated TAMSD. For example, when a normally distributed measurement error (with variance *σ* ^2^ and zero mean) is introduced to ergodic anomalous diffusion measurements, the theoretical TAMSD is [40, 41]

$$\bar{\delta^{2}}\left(\tau \right)=D_{\alpha }\tau^{\alpha }+2\sigma^{2}$$ (3)

For a discussion of the influence of various error mechanisms on the TAMSD of CTRW diffusion, see [42].

For normal diffusion various alternative and complementary techniques have been developed [39, 43] that overcome these problems. In addition, anomalous diffusion can be efficiently estimated when an ensemble of trajectories is available [41]. However, when analyzing single trajectories of particles that exhibit anomalous diffusion, these techniques are inadequate and one is left with the direct estimation of the functional form of the TAMSD.

When analyzing experimental data, one has a limited trajectory length and for single particle trajectories, it is often shorter than 10^3^ time points. This raises another fundamental problem in implementation of eqn. 2. Since the variance of the TAMSD increases with *τ*, taking large *τ*_*M*_ reduces the accuracy of the estimation. However, since the data is limited, the MSD also fluctuates at small *τ* values. In addition, as seen above, measurement errors introduce an offset at small *τ*’s. Thus one must find an optimal *τ*_*M*_ that balances between the need to fit several *τ*’s in eqn. 2 while avoiding the fluctuating nature of the TAMSD at large times. We stress that simply taking very small or large *τ*_*M*_ values does not improve the estimation of *α*, as can be seen in Fig. 1.

To the best of our knowledge, there is no systematic study of the optimal *τ*_*M*_ value for the estimation of the anomalous exponent in the presence of measurement errors. As a result, there are no standards or guidelines for fitting the TAMSD, which introduces difficulty in assessing the accuracy and precision of extracted values and comparison between studies. Furthermore, we show that the data analysis can be optimized by realizing the specific experimental conditions.

In what follows we study the performance of the TAMSD as an estimator for the anomalous exponent, depending on trajectory length, measurement error and the true anomalous exponent. This is done through the simulation of thousands of trajectories and fitting their individual TAMSDs. We study FBM diffusion, which we chose as an experimentally observed motion and a representative of the common class of ergodic anomalous diffusion [24]. We calculate the accuracy and precision of the TAMSD estimator as a function of the maximal fitted time lag, *τ*_*M*_, for different combinations of the diffusion parameters. The results allows us to identify an optimal ÏM in each case and by taking all the extracted information together, we identify several guidelines, or âbest practicesâ, for fitting of anomalous TAMSDs. We find that even a rough estimation of the measurement error and the expected regime of the anomalous exponent can greatly improve the accuracy of the extracted parameters.

Our approach can be applied to any process with a defined *α* that one can simulate in order to find the best estimation conditions, even if *D*_*α*_ varies between trajectories such as in CTRW or HDP. Although we focus on the more difficult experimental case of short trajectories, our general guidelines apply also for longer trajectories.

## Methods

Trajectories {*x*_*i*_(*t*)} were simulated using the MATLAB *wfbm* function [44] which is a common method for the simulation of fractional Brownian motion through a wavelet implementation, as proposed in [45]. In addition, we normalized the standard deviation of the increments for each trajectory to one, so that *D*_*α*_ = 1. Notice that FBM has stationary Gaussian increments, so normalizing the standard deviation uniquely defines the stochastic process for a given *α*.

A series of independent normally distributed measurement errors {*ε*_*i*_(*t*)} with zero mean and standard deviation *σ* was added to each trajectory. Since all trajectories were normalized, the relative magnitude of the measurement error compared to {*x*_*i*_(*t*)} is set only by *σ*. Also, note that for any uncorrelated measurement noise distribution that has a defined second moment, the magnitude of *σ* is enough to characterize its effect on the TAMSD.

We look into four representative cases of anomalous diffusion: *strong subdiffusion* *α* = 0.3, *weak subdiffusion* *α* = 0.7, *weak superdiffusion* *α* = 1.3 and *strong superdiffusion* *α* = 1.7. In each case, three error regimes are studied: low *σ* = 0.1, medium *σ* = 0.5 and strong *σ* = 1.

For each pair of *α* and *σ* we study trajectories of length *L* = 10 to 2000. For each trajectory we fit the TAMSD according to eqn. 2 for all possible *τ*_*M*_ up to *L*/2. We then repeat the calculation of the TAMSD and its fitting for 1000 trajectories for each *L* and *τ*_*M*_. Thus for each (*α*, *σ*) pair we have a set of *α*_*i*_(*L*, *τ*_*M*_) with *i* = 1, …, 1000 for every (*L*, *τ*_*M*_) combination.

We are now faced with the problem of identifying what is a ‘good’ fitting regime. One approach is to characterize the distribution of *P*(*α*_*i*_) for each (*L*, *τ*_*M*_) pair in each (*α*, *σ*) mapping. Then, one can estimate the probability of the fitted value to fall in a certain range around the true anomalous exponent. However, this approach is problematic as *P*(*α*_*i*_) is not necessarily normal. In fact, past studies have shown that the distribution of ⟨*δ* ^2^(*τ*)⟩ is highly non Gaussian [46, 47], leading to similar expectation for *P*(*α*_*i*_). As a result, analytically estimating probabilities will demand the characterization of general distributions.

Thus we take a different, more applicable approach where for each (*L*, *τ*_*M*_), we extract the fraction Φ_(*α*, *σ*)_(*L*, *τ*_*M*_) of *α*_*i*_ that are in the range [*α*−0.1, *α*+0.1]. We chose these limits since they provide reasonable accuracy in biophysical studies while maintaining reasonable Φ values for different (*α*, *σ*) maps. Φ is an intuitive parameter for the precision of the fitting, as higher values mean more precise fitting.

In some cases, one can extract multiple trajectories of the same stochastic process. This is for example the case in various simulation studies. Thus by averaging over fitted single particle *α*_*i*_’s, one may hope to converge with ⟨*α*_*i*_⟩ to *α*. We define the bias as *B*_(*α*, *σ*)_(*L*, *τ*_*M*_) = ⟨*α*_*i*_⟩_(*L*, *τ*_*M*_)_−*α*. This bias is a measure of the accuracy of the MSD estimator.

## Results

**Fig 2:**
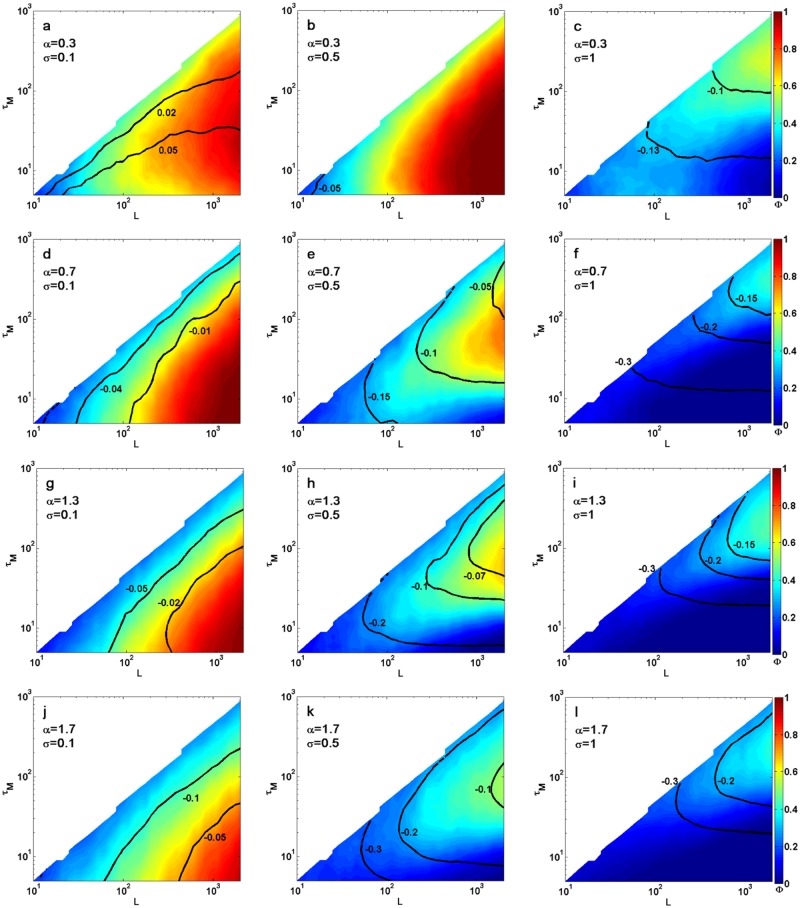
Performance of the time averaged MSD estimator for various trajectory lengths *L* and maximal time lags *τ*_*M*_. Color bar gives the precision Φ and black lines give representative bias values, *B*. Rows give various anomalous exponents with (a–c) strong subdiffusion *α* = 0.3, (d–f) weak subdiffusion *α* = 0.7, (g–i) weak superdiffusion *α* = 1.3 and (j–l) strong superdiffusion *α* = 1.7. Measurement error changes between columns with (left) small error *σ* = 0.1, (middle) medium error *σ* = 0.5 and large error *σ* = 1. The optimal *τ*_*M*_ is selected as the area where Φ is maximal and ∣*B*∣ is minimal for a given trajectory length *L*.

Fig. 2 shows a heat map of Φ with contour lines of the bias *B* for each measurement length *L* and *τ*_*M*_. As observed, it is easy to find the optimal *τ*_*M*_ for fitting, i.e. optimal Φ and *B* conditions. For example, for a thousand time point trajectory in the weakly subdiffusive regime (*α* = 0.7) with *σ* = 0.5, we find a maximal Φ ≈ 0.63 for *τ*_*M*_ = 50. In addition, 0 < *B* < −0.1 gives reasonable results for averaged TAMSDs. However, if the trajectory is only 100 time points long, it is best to use *τ*_*M*_ = 10, giving Φ ≈ 0.36 and −0.1 < *B* < −0.2.

We recommend the extraction of the optimal *τ*_*M*_ for each experiment depending on the exact conditions. Table 1, however, gives a quick look-up table for optimal *τ*_*M*_ for *L* = 100 and 1000 depending on *α* and *σ* and can be used to quickly analyze experimental data.

**Table 1:** Recommended *τ*_*M*_ values.

*α*	*σ*	*L*	Optimal *τ*_*M*_
0.3	0.1	100	15
		1000	20
	0.5	100	10
		1000	10
	1	100	20
		1000	200
0.7	0.1	100	10
		1000	10
	0.5	100	10
		1000	40
	1	100	40
		1000	400
1.3	0.1	100	10
		1000	10
	0.1	100	10
		1000	45
	1	100	40
		1000	150
1.7	0.1	100	10
		1000	10
	0.5	100	20
		1000	75
	1	100	50
		1000	150

We now describe several trends in the maps of Φ and *B*. The two fundamental observations are that lower *α* or *σ* usually give better estimation results with the TAMSD. This is expected according to equation 3 in [41], which shows that the estimation error is less significant at smaller *α* and *σ* values. Beyond this first order behavior, however, Φ and *B* show a rich picture depending on *α*, *σ* and *L*.

**Small measurement error**—when the experimental error is much lower than the average diffusion step, i.e. *σ* = 0.1, the small *τ* error of the TAMSD disappears, eqn. 3. In small *τ*’s there is less overlap between squared displacements leading to lower variation of the TAMSD. Indeed, for almost all *α* maps with *σ* = 0.1, we found that the best *τ*_*M*_ = 10, regardless of *L* (Fig. 2 left column). The one exception is for strongly subdiffusive motion, where *τ*_*M*_ = 20 is needed for large *L*’s.

In addition, a monotonous increase in optimal Φ is seen from a typical 0.4 when *L* ≈ 50 to Φ → 1 for *L* → 10^3^. The typical bias, *B*, is also usually better than −0.05, except for highly superdiffusive motion where 0 > *B* > −0.05 only for *L* > 10^3^. Thus in the regime of weak experimental error, TAMSD fitting of the first few *τ* can give good estimation of anomalous exponents. It is important to notice that for *α* = 0.3, *B* is positive, unlike other *α* values.

**Medium measurement error**—In the case of *σ* = 0.5, i.e. when the typical step size is twice the measurement error, the optimal *τ*_*M*_ changes with *L*, Fig. 2 middle column. With the exception of *α* = 0.3 we find that the best Φ is obtained when taking *τ*_*M*_ at 10–20% of short trajectories and 4–7% of long trajectories (higher values are for higher expected *α*). The values of Φ are lower than in the low localization error regime by a typical 0.2. In addition, caution should be used when averaging short trajectories, *L* < 10^2^ as bias can reach values worse than −0.2 for superdiffusive motion.

Interestingly, strong subdiffusive motion can be analyzed with *τ*_*M*_ = 10 to give better results than when *σ* = 0.1. This is possibly due to the measurement error lowering the extracted exponent and preventing high *α* values. Notice that if one uses the optimal *τ*_*M*_ that was found for *α* > 0.3, excellent results are still received for the strong subdiffusion case.

**Large measurement error**—When *σ* is the same size of the average particle step, accurate estimation of the anomalous exponent is hindered, Fig. 2 right column. For short trajectories, Φ values are approximately 0.2 and *B* ≤ −0.3. Thus if the measurement error is large, one should not estimate TAMSDs of short lengths *L* ≤ 300. Even for *L* ≈ 10^3^ we find values of Φ ≈ 0.4 with biases that can approach −0.15.

In fact, for *α* = 0.7 it is better to sub sample an *L* = 2⋅10^3^ trajectory every 7 time points giving an effective trajectory of *L* = 285 and *σ* = 0.5. Analysing this shortened trajectory with *τ*_*M*_ = 34 gives Φ = 0.49, compared to an optimal Φ = 0.42 obtainable from the original trajectory.

For strong subdiffusion, we find that best results are received when *τ*_*M*_ is 20% of *L*. However, the bias is still significant with *B* ≈ −0.1 for most conditions.

## Discussion

After identifying the trends and pitfalls in Φ and *B*, we now discuss the best practices for anomalous exponent estimation with the TAMSD. It is clear that with more knowledge regarding the regime of the anomalous exponent and the measurement error, a better decision of *τ*_*M*_ can be taken. We divide the recommendations into the following cases: a) perfect knowledge of *σ* and no necessary knowledge of *α*; b) approximate knowledge of both *σ* and *α*; and c) unknown *σ*. Finally we discuss the implications of having repeated realizations of the same process.

**Case a: Perfect knowledge**—If there is perfect knowledge of *σ* than a simple correction can be performed to bring the trajectory into the *σ* → 0 regime. Simply, for the analyzed TAMSD one should fit $\hat{\delta^{2}}(\tau )=\bar{\delta^{2}}(\tau )-\sigma^{2}$ to a power law. Even if there is no knowledge of the expected *α*, a limit of *τ*_*M*_ = 10 when fitting $\hat{\delta^{2}}$ will give the best results. Notice that if *α* is known to be strongly subdiffusive, it may be beneficial to take a slightly larger *τ*_*M*_.

**Case b: Approximate knowledge**—When the magnitude of the measurement error is only approximately known, the correction performed in case (a) will leave some residual *σ* > 0. If $\hat{\delta^{2}}(\tau =1)>\sigma^{2}>0$ we are in the regime of medium error. In such a case, knowledge regarding the expected *α* regime will help select the optimal *τ*_*M*_.

It is important to notice, that even if some measurement error is suspected but actually *σ* < < 0.5, the recommended *τ*_*M*_ values will not lower the expected Φ. Rather, Φ will usually increase with reduction in *σ* even for sub optimal *τ*_*M*_. The benefit of knowing that *σ* < < 0.5 is that one can take even more efficient *τ*_*M*_ values.

**Case c: Unkown *σ***—If there is no estimation of the measurement error, extraction of the anomalous exponent can lead to significant errors. Specifically, for short trajectories (*L* ≤ 300), the possibility that *σ* ≥ 1 leads to an inability to estimate *α*, unless the process is strongly subdiffusive (i.e. *α* ≤ 0.3). Since it is possible that *σ* > 1, Φ may be even lower than in the cases studied in this work. Thus, if the magnitude of *σ* is unknown, it is advised not to perform estimation of trajectories unless *L* ≥ 10^3^, and only if it can be assumed that *σ* is not significantly larger than unity.

For this reason, we advise that in all TAMSD studies an estimation of the measurement inaccuracy be given. Without this estimation, or the clear statement of its lacking, it is impossible to assess the anomalous exponent results.

**Multiple identical realizations**—In some studies, it is possible to extract many trajectories of the same stochastic process, where the underlying *α* is identical or comes from a narrow distribution around an average value. In such cases, one may average over many instances of the process, and Φ becomes irrelevant.

However, as we have seen, in cases of high measurement inaccuracy, *B* is still significant for many *L*’s. It is thus necessary to correct for the bias by adding an expected error factor to the extracted average exponent, ⟨*α*⟩. Another option is to study the large *τ* behavior of the particle averaged TAMSD $\left\langle \bar{\delta^{2}}(\tau )\right\rangle$, in the domain that is not affected by the measurement error or fit the particle averaged TAMSD directly to eqn. 3.

In biological and complex systems, this is usually not the case, and *P*(*α*) is widely distributed (a standard deviation of *σ*_*α*_ = 0.2 is considered wide). If the distribution can be approximated by a normal distribution, than $\left\langle \bar{\delta^{2}}(\tau )\right\rangle$ can be analyzed with previous techniques [41]. Otherwise single particle analysis is needed and the typical bias, *B*(*L*, *σ*, *α*) should be added to all trajectories based on *L* and some estimation of the anomalous diffusion regime.

Special care should be taken when estimating weakly non ergodic processes as the diffusion coefficient varies between trajectories, thus changing the relative size of *σ* [36, 48]. Since varying relative *σ* values leads to a varying bias in *α*, one will suffer a varying bias for each trajectory. Hence it may appear that *α* is distributed—in contradiction to the expectation for HDP and CTRW. Thus when characterizing weakly non ergodic processes with the TAMSD, one must strive to know the magnitude of the measurement error precisely.

## Conclusions

We have studied the efficiency of the MSD technique in the estimation of the anomalous exponent depending on the various underlying parameters. The main picture that arises is that the TAMSD is not an efficient technique when looking at short trajectories, or superdiffusive processes with non ideal measurements. When analyzing measured trajectories it is important to estimate beforehand the measurement error and the expected regime of the anomalous exponent. Then one must choose the maximal time lag, *τ*_*M*_, based on the efficiency of the MSD estimator and not according to a visual fit to the MSD. Importantly, for some experimental scenarios the TAMSD is highly inaccurate and it should not be used.

According to our findings, when specifying extracted parameters of anomalous diffusion processes, it is important to describe the means by which the specific fitting regime was selected including the expected accuracy and precision. This will enable to compare different experiments and more objectively validate proposed theories.

Finally we encourage the development of new estimation techniques for anomalous diffusion single particle trajectories. Without the advancement of theses techniques, the study of accurate anomalous exponents in complex experimental systems will not be possible.
